# Possibilities of Influencing the Crystallization Process of Bisphenol A- and Bisphenol F-Based Epoxy Resins Used for Hydrophobic Coatings on Concrete

**DOI:** 10.3390/polym15193871

**Published:** 2023-09-24

**Authors:** Michaela Seidlová, Jakub Hodul, Nikol Žižková, Ruben Paul Borg

**Affiliations:** 1 IN-CHEMIE Technology s.r.o., 779 00 Olomouc, Czech Republic; michaela.seidlova@in-chemie.cz; 2 Faculty of Civil Engineering, Brno University of Technology, 602 00 Brno, Czech Republic; zizkova.n@fce.vutbr.cz; 3 Faculty for the Built Environment, University of Malta, MSD 2080 Msida, Malta; ruben.p.borg@um.edu.mt

**Keywords:** epoxy resin, crystallization, coatings, mechanical parameters, glass transition temperature, bisphenol, chemical resistance

## Abstract

Crystallization of bisphenol A (DGEBA)- and bisphenol F (DGEBF)-based epoxy resins is a natural property of these oligomers. However, manufacturers of coatings and other systems based on these epoxy resins are making efforts to slow down the crystallization process as much as possible, thereby extending the shelf life and improving the competitiveness of their products. This paper focuses on the kinetics of the crystallization process of epoxy resins and the effect of the presence of a certain degree of crystallinity on selected parameters of epoxy-based materials. Furthermore, an analysis of the impact of a certain degree of crystallinity of the epoxy base on the resulting coating parameters was carried out. The highest value of crystallinity (17%) was achieved in the sample containing the highest proportion of DGEBF in the crystallization phase “c”, and the enthalpy of melting (H_t_) of the crystalline DGEBF sample was 6.3 J/g. Mechanical parameters as well as chemical and thermal resistance of hydrophobic epoxy systems were investigated. The best abrasion resistance (1.5 cm^3^/50 cm^2^) was achieved with the blend containing only amorphous DGEBA. The adhesion of the epoxy samples on concrete was greater than 6.5 MPa. The chemical resistance tests performed showed that, in general, the chemical resistance of epoxy systems decreases with increasing crystallinity content. The tighter arrangement of molecules in the crystalline regions of the epoxy matrix results in an increase in density, strength and hardness. This study presents a comprehensive examination of the crystallization of DGEBA and DGEBF, which is, as yet virtually unavailable. It also contributes to knowledge by outlining the possibility of speeding up or slowing down the crystallization process of epoxy resins, including the principle of selecting nucleating agents.

## 1. Introduction

The ever-increasing demands on the quality of epoxy-based coatings and flooring materials, as well as the unrelenting pressure on price, are the driving forces that lead the manufacturers of these products to continuously remodel their products in order to maintain their competitive edge in the market. The pressure for quality very often comes down to technological possibilities. Quality in the case of coating systems is not only related to the resulting mechanical and other performance parameters, but also shelf life. It is not just associated to the length of time that the product retains the declared parameters, but also storage conditions. Improperly stored products are harder to process and cause defects in the resulting surfaces. Epoxy-based coatings react to improper storage by crystallization of the epoxy base. A very unfavourable factor that has a critical effect on shelf life is the range of temperatures to which epoxy resin coatings are exposed (temperature variation in the warehouse between day and night, etc.). As this temperature interval increases, the kinetic conditions for crystallization become more favourable [1,2]. Currently, every effort is being made to slow down the crystallization process. So far, there are no known ways by which the occurrence of epoxy base crystallization can be eliminated [3]. Crystallization cannot yet be completely prevented. There are many influencing factors that contribute to the speeding-up or slowing-down of the crystallization process. It is advisable to take all of these factors into account and evaluate their partial effects when optimizing the storage life of an epoxy-based coating. The tendency of liquid epoxy resin to crystallize varies depending on factors such as system composition, purity, presence of additives, homogeneity, water content and other external factors [4]. It is very difficult to determine the tendency of liquid epoxy resin to crystallize. Thus, the tendency of epoxy resin to crystallize is expressed as the result of observing the change in fluidity and appearance of samples over time. Crystallization is a phenomenon that is difficult to simulate [5].

Generally, a depression in the growth kinetics of the crystallizable component is observed upon addition of the non-crystallizable component [6]. In polymer blends, where one component is crystalline and another one is amorphous, the curing of the second component results in chain extension, branching, crosslinking and significant changes in the chemical and physical properties of the non-crystalline component [7]. It has been reported that crosslinking can weaken the intermolecular hydrogen bonding and remarkably affect the crystallization morphology of blends [8,9]. By comparing the crystallization kinetics after and before the crosslinking reaction, Zheng et al. [10] found out that the curing reaction caused a considerable increase in the overall crystallization rate and that the mechanism of nucleation and the growth of the poly(ϵ-caprolactone) crystals were also dramatically changed. Resins and hardeners can all experience crystallization and it primarily occurs with two component systems, but can also occur in one-component heat-cured resins [11]. The crystallization temperature significantly affects the rate of crystallization, and for all crystallizable polymers, the temperature dependence is similar with a maximum between the melting temperature and the glass transition temperature of the polymer under investigation. If the crystallization temperature lies in the region close to the melting temperature, the rate of crystallization is very small. When the temperature decreases, the crystallization rate increases rapidly [12]. During polymer processing, various additives like color batches, nucleating agents and fillers are used. Such additives may influence the crystallization kinetics and the structural formation in the polymer [13]. One of the most common nucleating agents in thermoplastics and also in reactoplastics, especially polyesters, is talc, which crystallizes in a monoclinic crystallographic system [14].

Crystallization germs are formed in the polymer mainly as a result of local temperature fluctuations and are formed by associated chain segments. We can distinguish between isothermal and non-isothermal crystallization. A germ formed at an isothermal temperature has comparable dimensions in all directions and grows uniformly in all directions right from the start. A radial spherulitic spherulite is formed. The germ formed at a non-isothermal temperature prefers to grow in the direction of its lamellae, branches and forms a bundle, which continues to grow and expand until it is closed. Growth continues uniformly in all directions. A spherulite with a tuft-like structure called a dendritic spherulite or dendrite is formed [15]. Dendrites are formed during rapid crystallization [16].

Epoxy resins, acrylics and polyurethane are traditional protective hydrophobic coatings which have been used in construction for many years [17]. Hydrophobic agents are surface protection materials capable of increasing the angle of contact between the water and the concrete surface by reducing water (in liquid form) penetration in concrete [18]. Concrete hydrophobicity is an important index that is closely related to concrete durability [19,20]. Epoxy coatings and siloxanes have been found to be very effective in reducing the water uptake of concrete [21]. Hydrophobizations should penetrate into the substrate as deeply as possible to ensure the long-term durability of concrete [22]. Numerous pure and cured ERs are evaluated as effective anti-corrosive materials for different metals and alloys in different electrolytic media especially in neutral sodium chloride solution [23].

In quite a few epoxy resin formulations, fillers are necessary. Fillers improve mechanical properties such as strength, stiffness and modulus [24]. Epoxy resin based on diglycidyl ether of bisphenol A (DGEBA) is most often brittle and not resistant to cracking; therefore, it is reinforced with various fillers and fibers in order to produce a material with higher strength [25]. The modification of epoxy resins with nanoparticles could endow the materials with some superior properties such as an increase in the glassy modulus, broadening of the glass transition temperatures and significant improvement in the main mechanical properties [26].

This paper deals with the analysis of the impact of a certain degree of crystallinity of epoxy base on the resulting parameters of the hydrophobic coating, such as mechanical parameters, as well as the chemical and thermal resistance of coatings. The aim was to find a way to better understand the significance of the different influencing factors on the crystallization of epoxy-based coating systems, as well as to find new possibilities in the control of the crystallization process in these systems and, eventually, to use crystallization in the preparation of coating systems based on modified epoxy resins. In fact, based on experience from the behavior of other semi-crystalline polymers, we know that due to the tighter arrangement of molecules in the crystalline regions of the matrix, the density, strength, modulus and hardness are increased.

First, it was necessary to effectively simulate the crystallization process in order to be able to compare in a relevant manner the tendency of different epoxy resins and modified mixtures to experience crystallization. The formulations were designed in such a way that there was a tendency for crystallization to occur for both pure DGEBA and DGEBF epoxy resins, as well as various combinations of selected nucleating agents and substances that theoretically slow down nucleation, or the crystallization process of epoxy resins. Furthermore, the degree of crystallinity of the samples, glass transition temperature (T_g_) and other parameters were determined using DMA analysis. An analysis of the impact of a certain degree of crystallinity of the epoxy base on the resulting parameters of the coating was also carried out. Both the mechanical parameters and the chemical and thermal resistance of the coatings were tested.

The contribution of this paper is also an outline of the possibility of speeding up or slowing down the crystallization process of epoxy resins and coating systems produced from them, including the principle of selecting nucleating agents. Furthermore, the method of slowing down the crystallization process of DGEBA/DGEBF mixtures has been described in detail, which is known, but the specific dependence of the DGEBF content on the crystallization rate is still unavailable. This information has a major impact on the manufacturer. Testing for crystallization tendency is very time-consuming and tedious. The correct ratio of DGEBA/DGEBF ensures a long life of the resulting system. At the same time, however, it is inappropriate to set the dosage of DGEBF too high due to the high price of DGEBF compared to DGEBA. The experimental verification of the optimal dosage of DGEBF in the DGEBA/DGEBF mixture is a great benefit of this research. The results describe in detail the various mechanical, chemical and thermal resistances of amorphous and semi-crystalline DGEBA/DGEBF systems cured with an isophoronediamine-based crosslinker. The presence of crystallinity was found to increase the hardness and brittleness of the resulting polymer.

## 2. Materials and Tested Formulations

### 2.1. Materials

Heterogeneous nucleation occurs if a polymer substance contains wettable impurities. The crystallization process can theoretically be slowed down by slowing down the movement of epoxy resin molecules. The formulations were designed in such a way as to detect the tendency to crystallization of both pure DGEBA and DGEBF epoxy resins and reactive thinners, as well as various combinations of selected low-molecular non-reactive thinners, thixotropic additives and fillers corresponding to the theoretically possible content of the most common additives in epoxy coating systems; alternatively, they were designed by verifying the theoretical assumptions of the effect of admixture on the speed of the crystallization process. Compared to unmodified bisphenol A epoxy resin, bisphenol F epoxy resin has a much lower viscosity, higher epoxy index and higher epoxy functionality. Benzyl alcohol and reactive thinners up to a total content of approx. 5–10 wt.% usually have little effect on the properties of the resulting system. The choice of materials that can be used as a filler is limited by already known properties and limits. The main limiting factor is based on the chemical incompatibility between the filler and the base matrix. The specific weight of the filler must be higher than the specific weight of the binder base in order to prevent leaching of the fillers from the matrix, but at the same time it must not be significantly higher due to the subsequent higher tendency for the filler to sediment in the matrix.

#### 2.1.1. Unmodified Epoxy Resins Based on Bisphenol A (DGEBA) and Bisphenol F (DGEBF)

These are clear, transparent, liquid, low molecular weight, unmodified resins intended for the production of composite materials, hand lamination, pultrusion, coating chemistry, etc. The representative for DGEBA resin was DER 331, and for DGEBF resin it was DER 354 manufactured by Palmer Holland (Westlake, OH, USA). When choosing epoxy resins, their commercial availability in the Czech market and quality were taken into account. Basic properties of the epoxy resins are stated in Table 1.

#### 2.1.2. Thixotropic Additive–Pyrogenic Silica (PS)

Thixotropy is a rheological property of some pseudoplastic and plastic systems, e.g., epoxy coatings, which, when subjected to shear stress (mixing, shaking, etc.), initially exhibit a high apparent viscosity that gradually decreases with time. If the system is left at rest, the original structures are restored and the viscosity asymptotically approaches the original high value [29]. It is synthetically produced from pure SiO_2_ and is produced both as untreated (pyrogenic) and treated (hydrophobic). The fumed silica is produced by a chemical treatment of hydrophilic grades with silanes or siloxanes. In the finished product, the treatment agent is chemically bonded to the former hydrophilic oxide. It is characterized, among other things, by its low moisture adsorption, excellent dispersibility and ability to adjust rheological behavior, even that of polar systems [30]. Two types of pyrogenic silica (PS) from the manufacturer EVONIK (Essen, Germany), with a specific surface area of 200 m^2^/g and with a specific surface area of 380 m^2^/g, were selected for the research. Both types contained particles having a maximum size of 50 μm.

#### 2.1.3. Fillers

Powdered materials such as barite, silica flour, etc., are most often used as fillers. Epoxy-based hydrophobic coating systems are particulate composite materials. The following fillers were selected for the research:

- Baryte;
- Waste windshield glass;
- Silica flour Dorsilit.

Waste windshield glass imported from a landfill in the Czech Republic was first broken, including the foil, and then the individual pieces were ground in a ball mill to separate the glass from the polymer foil, which was removed. The grinding took a total of about 1 h with a weight of 3 kg of shards. Next, the ground glass was manually sieved to a granulometry ≤ 0.063 mm. The glass thus prepared had a density of 2500 kg/m^3^. The chemical composition of the waste glass was the following: 69.2% SiO_2_, 12.0% Na_2_O, 9.2% CaO, 3,7% MgO, 0.7% Al_2_O_3_, 0.3% K_2_O, 0.2% Fe_2_O_3_.

Two types of silica sands (flours) were selected, namely Dorsilit 1600 and Dorsilit 12100. Dorsilit 12100 is a type of silica flour with a particle size < 63 μm, and Dorsilit 1600 was chosen as the type of silica flour with a maximum particle size > 63 μm. The reason for this selection was to determine the effect of particle size on the rate of the crystallization process. Silica sands with grain sizes ≥200 μm are not used as primary fillers due to rapid sedimentation in the polymer matrix due to their large particle size. These fillers are used as secondary fillers, which are mixed in just before application. Secondary fillers have no effect on the crystallization process. Their individual properties, including their producers, are listed in Table 2.

#### 2.1.4. Solvents

Pure bisphenol A-based epoxy resins have a viscosity at +20 °C typically in the range of 10,000 to 15,000 mPa·s. and pure bisphenol F-based epoxy resins have a viscosity at +20 °C typically in the range of 5000 to 7000 mPa·s. The viscosity of both types of resins is unsuitable for most processing applications. Using diluents, whether reactive or non-reactive, it is possible to reduce the viscosity of the newly formed mixture to about 500 mPa·s at +20 °C. Non-reactive diluents have the disadvantage of odor and the fact that even at lower dosages, from about 1 wt.%, they can have a significant negative effect on the resulting mechanical, chemical and heat resistance of the polymer.

From the non-reactive diluents, two were selected, being the most commonly used in the formulation of solvent-free epoxy systems. Their relative representation in the final system is usually very small. They are not usually added to systems on their own, but almost always as part of additives to modify various properties of the final system or as part of modified amine hardeners. Non-reactive diluents flow out of the system throughout the life of the solvent-free epoxy coating. If they are dispensed in large quantities, their leaching can have a very negative effect on the life of the coating. Increased dosage of non-reactive thinners most often results in paint shrinkage. Increased coating shrinkage results in cracking and peeling of the film from the substrate. Typical properties of used reactive and non-reactive solvents are stated in Table 3.

#### 2.1.5. Nucleating Agents

Nucleation is a process by which nuclei begin to form in the melt, and subsequently, lamellae and later spherulites are formed from these nuclei. Minerals have proven to be one of the most effective nucleating agents in the field of polymer crystallization. In the conducted research, precipitated calcium carbonate according to ISO 4895 was chosen as the nucleating agent. Calcium carbonate crystallizes in the rhombic crystallographic system. If a crystalline substance successfully functions as a nucleating agent in a certain matrix, then there is a high theoretical probability that other substances crystallized in the same crystallographic system can also be suitable nucleating agents for the same matrix. Baryte was chosen as a suitable and available mineral from the same crystallographic system. Its influence on the nucleation and crystallization process was partially tested, as baryte was also used as a filler.

#### 2.1.6. Tested Formulations

The formulations for the determination of tendency to crystallize according to EN ISO 4895 standard [31] are stated in Table 4. The formulations were designed to determine the tendency to crystallize of both pure DGEBA and DGEBF epoxy resins and reactive diluents, as well as various combinations of selected low molecular weight non-reactive diluents, thixotropic additives and fillers from the theoretically possible content of the most common admixtures of epoxy coating systems, or to verify the theoretical assumptions of the influence of admixtures on the rate of the crystallization process.

The degree of crystallinity was ensured by adding the target crystallized resin DGEBF type DER 354 to the amorphous resin DGEBA type DER 331. The epoxy bases were then cured with an isophorone diamine-based crosslinker. The formulations for determining the mechanical parameters, chemical resistance and glass transition temperature of the selected epoxy systems are shown in Table 5. The formulations were formulated in such a way that the results of the tests captured the degree of crystallinity of the epoxy resin from maximum to zero crystallinity, thus allowing for the most accurate description of the influence of a certain degree of crystallinity on the mechanical, chemical and thermal parameters of the final polymerized system. Many parameters of solid polymers are predetermined by their degree of crystallinity [32,33].

## 3. Methods

### 3.1. Determination of Tendency to Crystallize

The test was performed according to the standard EN ISO 4895 [31]. The tendency of liquid epoxy resins to crystallize varies depending on various factors such as composition, purity, additives, homogeneity and water content. Furthermore, external factors such as storage history or room temperature have a significant influence. It is very difficult to quantify the tendency to crystallize. Therefore, it was determined by observing and comparing the fluidity and appearance of the samples. The principle of the method is that calcium carbonate powder is mixed into a liquid epoxy resin which is diluted with ethanol. This mixture was then kept at low temperatures and changes in fluidity and crystallization were observed, at intervals.

**Figure 1:**
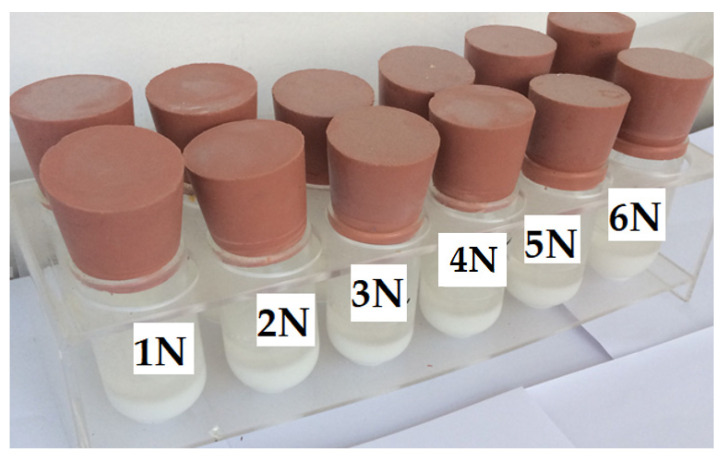
Set of samples for determining the tendency to crystallize according to ISO 4895 standard.

**Figure 2:**
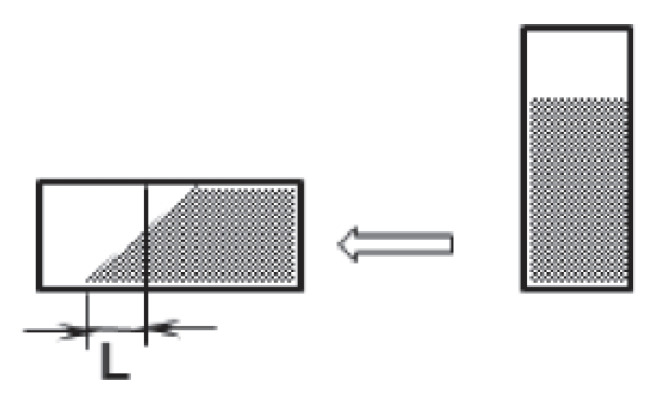
Evaluation of samples in determining the tendency to crystallize.

The principle of the test consisted of injecting 20 g of epoxy resin or epoxy mixture into a glass tube with a volume of 100 mL, having a 40 mm diameter and being 80 mm high (see Figure 1). The tube was then sealed with a special rubber stopper and placed in a thermochamber heated to (60 ± 2) °C for 16 h. The tubes were then removed from the thermocouple and allowed to cool spontaneously to room temperature (23 ± 5) °C. Nucleating reagents were added to the tubes according to the formulation stated in Table 4 and the mixture was stirred with a glass rod. Some samples were left without nucleating reagents and so were just mixed with a glass rod in this step. The tubes were sealed with a rubber stopper and placed vertically in a refrigerator at an internal temperature of (10 ± 2) °C. The samples were evaluated twice a day at intervals of 8 h during the day and 16 h during the night. The evaluation was performed by removing the tubes from the refrigerator, allowing them to warm spontaneously to room temperature (23 ± 5) °C, and then placing them in the horizontal position for 1 min as shown in Figure 2.

After each evaluation, the contents of the tube were mixed for 2 min with a clean glass rod, and then the tube was sealed with a rubber stopper and placed back in the refrigerator. The differential scanning calorimetry (DSC) and the scanning electron microscopy observations showed that there were large differences in crystallization and morphology between samples prepared by mixing in a different sequence [34]. Therefore, the mixing procedure must be followed thoroughly. The value of L should be thoroughly evaluated and recorded. If the level of the tube contents does not change when placed horizontally, i.e., it is completely vertical, then the sample has crystallized, the crystallization phase is marked “c” and the sample is discarded from further observation. The evaluation of the crystallization phase was performed as follows:

- L ≥ 10 mm—the crystallization phase of the sample is evaluated by the letter “a”;
- L < 10 mm—the crystallization phase of the sample is evaluated by the letter “b”;
- L = 0 mm—the crystallization phase of the sample is evaluated by the letter “c”.

### 3.2. Determination of Crystallinity

Determining the crystallinity of the prepared samples is a very important step, that is necessary to ensure the reproducibility of the test results. At the beginning of the research, the plan was to measure crystallinity through differential scanning calorimetry (DSC). However, further study of the issue revealed that this procedure cannot be applied. It was experimentally verified (see Section 3.3. DSC analysis) that the absolute enthalpy of H_0_ of crystalline DGEBA and DGEBF is a completely abstract concept. Epoxy resins based on DGEBA and DGEBF are not able to go completely into the crystalline phase. A certain amorphous part always remains in the volume, which makes it impossible to determine the absolute enthalpy H_0_. In a subsequent step, the possibility of using X-ray analysis to determine the degree of crystallinity of the samples was considered. However, even this method had to be ruled out as a possible approach. The melting points of epoxy resins based on DGEBA and DGEBF are below normal room temperatures. Equipment for determining the crystallinity of samples with a melting temperature below +20 °C by X-ray analysis was not available. After further study of the professional literature, it was decided to determine the crystallinity through the calculation method. The calculation of the crystallinity of the samples was performed according to Equation (1) for calculating the crystallinity of semicrystalline polymers, where X is the crystallinity (takes on values from 0 to 1), ρ is the specific weight in units of kg/L, ρ_c_ is the specific weight of solid (crystallized) epoxy resin and ρ_a_ is the specific weight of amorphous epoxy resin.

(1) $$\rho =X\times \rho_{c}+(1-X)\times \rho_{a}$$

The specific gravity of the selected samples was determined using a pycnometer with a volume of 50 mL. For the purposes of this analysis, a sample of spontaneously crystallized DGEBF epoxy resin was used as the solid (crystallized) epoxy resin. DGEBA epoxy resins were not included, as it was not possible to prepare solid (crystalline) resin samples by accelerating the crystallization process according to ISO 4805 at this stage. A sample of the spontaneously crystallized DGEBA epoxy resin to phase “c” according to ISO 4898, i.e., a solid (crystalline) sample, was also not available. The crystallinity of the samples listed in Table 5 was determined by calculating the value from the difference in specific weights of an amorphous, spontaneously maximally crystallized sample of DER 354 and a purposefully accelerated crystallized 10 kg sample of DER 354 in the crystallization phase “c”. In the case of the last of the mentioned samples, the crystallization phase “c” according to ISO 4895 was achieved by homogeneous inoculation of the “microseeds” type.

### 3.3. DSC Analysis

From a physical point of view, crystallization is an exothermic process. If the crystallization temperature lies in the region close to the melting temperature, the rate of crystallization is very low. As the temperature is lowered, the rate of crystallization increases rapidly. At the same time, the viscosity of the polymer melt also increases, which, together with the decrease in the kinetic energy of the macromolecules, causes the crystallization process to slow down, and therefore, after reaching its maximum, the rate of crystallization decreases with decreasing temperature. Near the glass transition temperature (T_g_), crystallization ceases as the movement of chain segments is severely restricted. Differential scanning calorimetry (DSC) is a thermal analysis in which the sample being monitored is subjected to linear heating or cooling while the rate of heat flow in the sample is continuously varied in proportion to the instantaneous specific heat. DSC is used to obtain characteristic temperatures such as melting and glass transition temperatures, or specific heat of fusion, crystallinity, control of annealing or curing. DSC analysis was performed on two samples of DGEBF resin—amorphous and spontaneously crystallized. The glass transition temperature of the DGEBF epoxy resin was very low and could not be reached in the measurements taken. A DSC 2500 device (TA Instruments, New Castle, DE, USA) in modulated mode was used for the measurements. The sample and the reference were always placed directly into the chamber in which the temperature was maintained at 0 °C. The following measurement method was applied: (1) ramp 5 °C/min to −20 °C; (2) temperature modulation on with an amplitude of 0.8 °C and a period of 20 °C; (3) duration of 3 min; (4) ramp 3 °C/min to 120 °C; and (5) duration of 6 min.

### 3.4. Determination of Viscosity

Dynamic viscosity of the epoxy systems was determined according to the standard EN ISO 2884-2 [35]. The principle common to all types of rotational viscometers is the measurement of the moment of force that must be overcome by a rotating body immersed in a liquid. The determination of the viscosity of the samples from Table 5 was carried out on a Myr VR 3000 rotational viscometer (Madar Yara Co., Riyadh, Saudi Arabia) using spindle R3, a rotational speed of 5 rpm and a sample temperature of (25 ± 1) °C. These measurement conditions, or spindle and rotational speed, are suitable for measuring viscosities of liquids in the range 3000 to 20,000 mPa·s. Only the viscosity of the epoxy resin mixture without the addition of isophorone diamine-based hardener was measured. This is due to the initiation of polymerization immediately upon mixing the hardener with the epoxy resin and the increase in viscosity due to the crosslinking of the molecules.

### 3.5. Determination of Hardness Shore D

Hardness of epoxy blends was determined in accordance with the standard EN ISO 868: Plastics and ebonite—Determination of indentation hardness by means of a durometer (Shore hardness) [36]. The principle of the test is the measurement of the depth of indentation of a specific indenter pressed into a sample under specified conditions. To determine the hardness, samples from Table 5 were prepared and cast into 10 mm thick Petri dishes and polymerized for 7 days at (20 ± 3) °C. The hardness of each sample was then measured using an analogue hardness tester type TQC, model LD055 (TQC Sheen B.V., Capelle aan den Ijssel, The Netherlands).

### 3.6. Abrasion Resistance

The abrasion resistance test, on specimens with dimensions of (71 ± 1.5) mm and a thickness of 20 mm, was carried out according to the EN 13892-3 standard [37]. The sample was placed in an N-1001 RT Böhm abrasion resistance device (FORM + TEST Seidner & Co. GmbH, Riedlingen, Germany) on a grinding track. The artificial corundum was used as abrasive material and the specimens were tested in 16 cycles of 22 rotations each, with a sample load of 294 N.

### 3.7. Glass Transition Temperature (T_g_) Determined by DMA

Dynamic mechanical analysis (DMA) belongs to the group of thermal methods. However, it is one of the most sensitive techniques capable of characterizing and interpreting the mechanical behavior of the polymerized material. DMA is based on monitoring the viscoelastic response of a material subjected to a small oscillatory stress. The deformation of the specimen is caused by two opposing moments of equal magnitude acting on opposite ends of the clamped specimen. Using DMA, the material can be characterized by the dependence of modulus and loss angle on temperature or time. Five representative formulations of epoxy thermosets were selected to determine the glass transition temperature (T_g_). These were formulations 5 M, 11 M, 13 M, 14 M and 15 M from Table 5. The epoxy blends were selected to represent samples containing only single amorphous and semicrystalline DGEBA and DGEBF, and then two samples containing amorphous DGEBA and amorphous or semicrystalline DGEBF in a 1:1 ratio. The measurements, carried out on a TA Instruments DMA 2980 (TA Instruments, New Castle, USA), were carried out on beams polymerized for 7 days at (20 ± 3) °C. First, the samples were heated to +35 °C, held at this temperature for 1 min, then the samples were heated to +120 °C at a rate of 3 °C/min. Due to the low initial measurement temperature, the evaluation of the decrease in the elastic modulus is very accurate.

### 3.8. Adhesion on Concrete

According to Table 5, specimens were made and applied to vibrated concrete pavements with a thickness of 3 cm and dimensions of 30 × 30 cm. The concrete substrate was sanded with sandpaper and acidified midway before application. The samples were applied by brush at a consumption of 1 kg/m^2^, i.e., a layer thickness of ~1 mm, and left to polymerize at (20 ± 3) °C for 28 days. In order to determine the adhesion according to EN ISO 4624 [38], metal dollies with a diameter of 20 mm were first glued to the surface of the coating. After 24 h, the dollies were fixed in an Elcometer 510 automatic pull-off adhesion gauge (Elcometer Ltd., Manchester, UK), and tensile stress was gradually applied at a rate of 1.00 MPa/s. At the moment of adhesion failure, the adhesion tester recorded the value of the adhesion. An important factor in evaluating the adhesion of the coating to the concrete is that the cohesion of the substrate of the coating should be greater than or equal to the adhesion of the coating.

### 3.9. Tensile Properties

Determination of tensile properties and specimen preparation were based on EN ISO 527-1 [39] and EN ISO 527-2 [40]. The samples were left at (20 ± 3) °C for 28 days before testing. The thickness of the dog bone type 1BA test specimens were 4 mm. From the measured values, the tensile strength and the relative elongation ɛ at first break were determined. The tensile test machine Testometric MT 350-20CT (Testometric Ltd., Rochdale, UK) was used for the testing of tensile properties, and three samples from each mixture were tested to determine the average value.

### 3.10. Determination of Chemical Resistance

After weighing and mixing all the components of samples 1 M, 6 M, 7 M, 12 M, 13 M, 14 M and 15 M according to the formulations given in Table 5, the prepared samples were applied with a clean brush at 20 ± 2 °C on acetone-cleaned microscope slides of 76 × 26 mm. After application, the samples were left to polymerize at (20 ± 3) °C for 7 days. The samples were then weighed to the nearest 0.01 g. The samples were immersed in glass cuvettes with the prepared chemicals, which are reported in Table 6. The samples were then checked after 24 h, after 7 days and after 4 weeks. Three parameters were checked, namely sample weight, adhesion to substrate and appearance. The weight loss (degradation–dissolution) of the sample or conversely the weight gain (degradation–swelling) are important indicators of the chemical resistance of any epoxy coating. A photographic record of the visual control was always taken.

## 4. Results and Discussion

### 4.1. Determination of Tendency to Crystallize

From the crystallization tendency analyses performed, it was found that DGEBA resins are less initiable for the crystallization process when compared to DGEBF. The evaluation of the determination of tendency to crystallize is reported in Table 7. The monitoring of tendency to crystallize was carried out twice a day, namely in the morning (M) at 7 a.m. and in the afternoon (A) at 5 p.m.

From the results of this analysis, it was found out that the DGEBF-based epoxy resin crystallized most readily and was the only one to reach the ISO 4895 crystallization stage “c” within 28 days. The next sample that reached at least the ISO 4895 crystallization phase “b” was the DGEBA epoxy resin DER 331 after 13 days. The DGEBF epoxy resin reached the crystallization phase “b” after only 4 days. No sample of DGEBA epoxy resin reached crystallization phase “c” according to ISO 4895 within 28 days of the start of the analysis. According to the analysis, low molecular weight substances, i.e., reactive and non-reactive diluents, have no significant effect on the rate of crystallization or the initiation of the crystallization process. In contrast, for thixotropic additives and fillers, an effect was observed in several cases. The influence of fillers and thixotropic additives, generally solid heterogeneous particles of the system, is particularly evident at lower concentrations. Although the solid heterogeneous particles in the system act as crystallization nuclei, they only act up to a certain concentration. Above about 30–40 wt.% of solid heterogeneous particles, their proportion in the epoxy matrix is so high that they begin to spatially hinder the folding of oligomeric chains of molecules into the crystal structure and thus slow down the crystallization process, in the initiation of which they themselves participated, quite substantially. This phenomenon can therefore also be observed from the results of the analysis of the tendency to crystallize samples containing heterogeneous solids. Samples containing smaller particles up to 63 μm in size, such as pyrogenic silica, barite and fine silica flour Dorsilite 12100, reached the ISO 4895 crystallization phase “b” within 28 days of the start of the analysis. On the other hand, the sample with filler particles above 63 μm (11 N) did not reach the “b” crystallization phase within 28 days of the start of testing. From the test results, the effect of surface structure on the rate of the crystallization process can also be seen. Although the other fillers and thixotropic additives used at lower dosages with particle sizes up to 63 μm accelerated the crystallization process to the extent that the samples reached crystallization stage “b” within 28 days of the start of the tests, this was not the case for the samples with the addition of the waste windshield glass-based filler. Furthermore, the dependence that the specific surface area size has an effect on the rate of the crystallization process or the rate of nucleation, was also confirmed. The larger the specific surface area of the heterogeneous particle, the faster the nucleation. Sample 7 N contained pyrogenic silica with a specific surface area of 200 m^2^/g and sample 8 N contained pyrogenic silica with a specific surface area of 380 m^2^/g. A faster crystallization process was observed for sample 8 N. The sample reached the crystallization phase “b” 4 days earlier than sample 7 N.

### 4.2. Determination of Crystallinity

**Figure 3:**
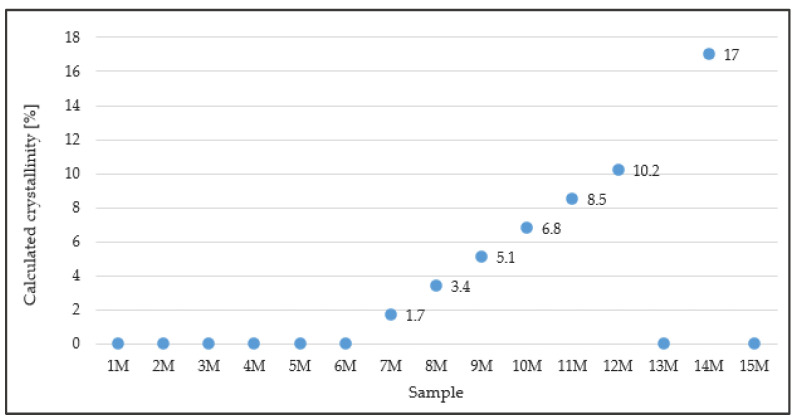
Graphical evaluation of the calculated crystallinity.

A graphical evaluation of the calculated crystallinity values of individual samples 1 M to 15 M, which were calculated on the basis of the input values listed in Table 8, is shown in Figure 3. As could be expected, the samples that contained the amorphous DGEBA and DGEBF resins showed zero crystallinity. The highest value of crystallinity (17%) was achieved in sample 14 M, which contained the highest proportion of DGEBF in the crystallization phase “c”.

### 4.3. Determination of Crystallization Temperature by DSC Method

**Figure 4:**
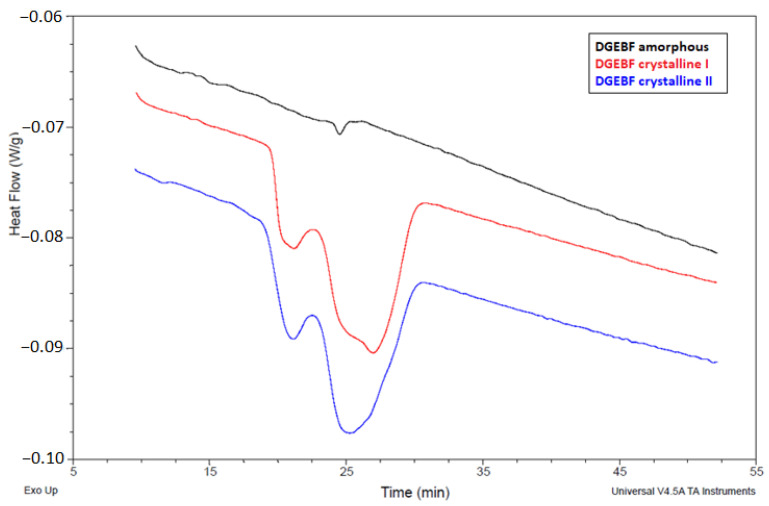
Graphical record of the dependence of the heat flow on time from DSC analysis.

**Figure 5:**
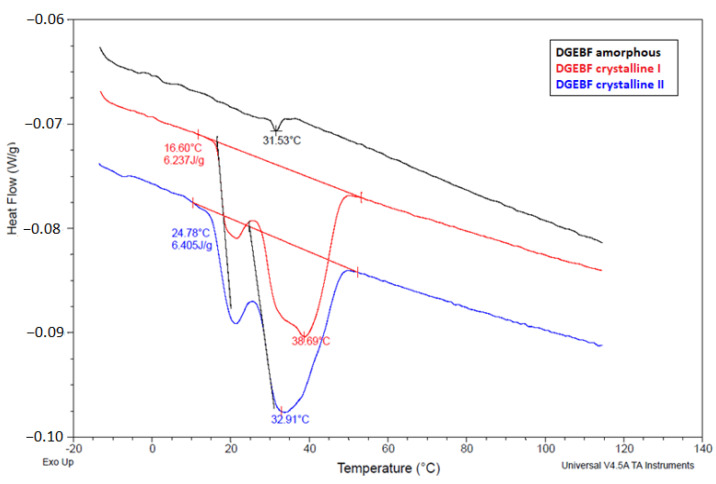
Graphical record of heat flux versus time from DSC analysis.

The glass transition temperature (T_g_) of the DGEBF epoxy resin was very low, so it could not be reached in this measurement. In this measurement, it was shown that two phases, crystalline and amorphous, coexisted in the DGEBF sample in crystallization phase “c” according to ISO 4895. The theoretical assumption that epoxy resins do not reach the phase of complete transformation from the amorphous to the crystalline state and, therefore, that the absolute H_0_ enthalpy of crystalline DGEBA and DGEBF is a completely abstract concept (therefore, the DSC method cannot be used to determine crystallinity), has been experimentally supported. The graphical record of the dependence of the heat flow on time for the DGEBF mixtures can be seen in Figure 4. The average observed enthalpy of melting H_t_ of the crystalline DGEBF sample was 6.3 J/g. From Figure 5, it is clear that the melting of the crystalline phase of the solid DGEBF sample starts around +15 °C and has a two-step progression indicating the presence of two structures. In Figure 5, a small endothermic peak can be seen in the amorphous DGEBF sample at 31.5 °C. This is most likely due to the relaxation motion of the molecules.

### 4.4. Determination of Viscosity

**Figure 6:**
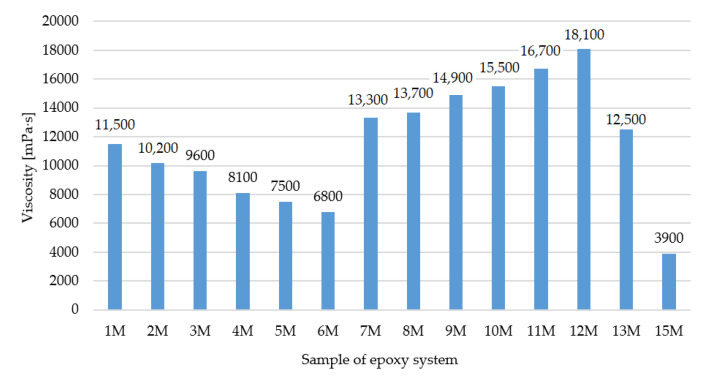
Dynamic viscosity of the epoxy mixtures.

The viscosity values shown in Figure 6 demonstrate that with increasing DGEBF epoxy resin content in the crystallization phase “c”, there was a significant increase in the viscosity of the entire DGEBA/DGEBF blend while the applicability of the samples deteriorated. 

### 4.5. Determination of Hardness Shore D

**Figure 7:**
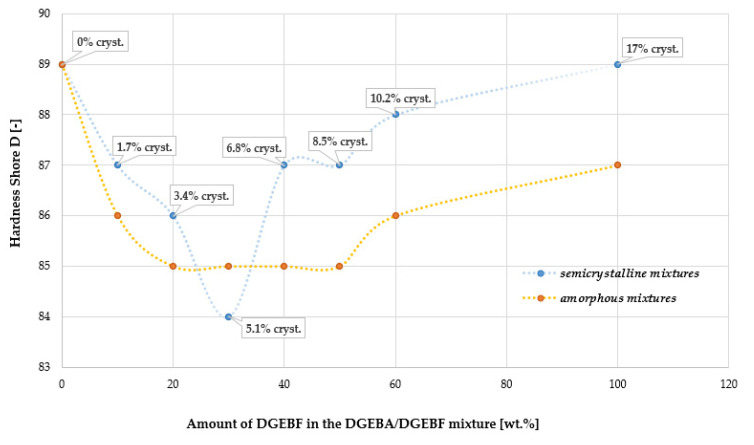
Dependence of Shore D hardness on crystallinity of DGEBF resin in different DGE-BA/DGEBF resin blends compared in mixtures with the same DGEBA/DGEBF ratio but zero crystallinity. Orange curve—amorphous mixtures, blue curve—semi-crystalline mixtures (crystallinity content introduced by DGEBF is indicated).

In the amorphous DGEBA/DGEBF mixtures, a decrease in Shore D hardness can be observed in Figure 7 for DGEBF containing mixtures in the interval 10–50 wt.%. For the crystalline DGEBA/DGEBF blends, there is a significant decrease in Shore D hardness around 5% crystallinity by 1 degree Shore D below the values of the amorphous blends. In general, the Shore D hardness is higher for semi-crystalline mixtures. Low crosslink density decreases the Shore D hardness [41].

### 4.6. Abrasion Resistance

**Figure 8:**
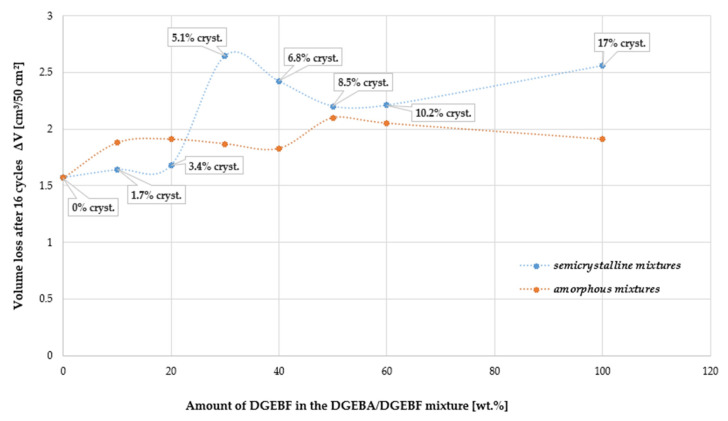
Dependence of Böhm abrasion resistance on crystallinity of DGEBF resin in different DGEBA/DGEBF resin mixtures.

From the dependencies in the graph in Figure 8, it is clear that the isophorodiamine-based hardener-cured polymer system containing only DGEBF as the epoxy resin in the ISO 4895 crystallization phase “c” exhibited lower abrasion resistance than the same system containing only amorphous DGEBF epoxy resin. On the other hand, when the DGEBF content of the DGEBA/DGEBF blend was 10–20 wt.%, it can be seen from Figure 8 that the crystalline versions of these blends had better abrasion resistance than the amorphous versions. This trend changes at 30 wt.% DGEBF in the DGEBA/DGEBF blend. This content of semi-crystalline DGEBF corresponds to ~5% crystallinity and a step deterioration in abrasion resistance occurred. The Shore D hardness result corresponds with this data, as it was found that there was a clear significant decrease in Shore D hardness around 5% crystallinity for crystalline DGEBA/DGEBF blends. The results of Shore D hardness and abrasion resistance show that there was a deterioration in mechanical parameters at crystallinity of DGEBA/DGEBF epoxy resin blends (DGEBA amorphous, DGEBF semi-crystalline) around 5%.

As the crystallinity increased further, the abrasion resistance improved to the same level as the equivalent amorphous DGEBA/DGEBF blends. The difference in values between the amorphous and semi-crystalline blends then occurred, as already mentioned, only at 100% DGEBF content on the epoxy resin side of the blend.

### 4.7. Glass Transition Temperature Determined by DMA

**Figure 9:**
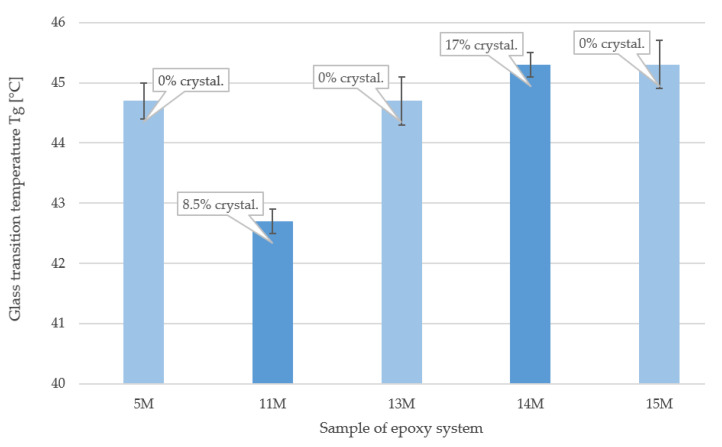
Results of T_g_ of specific samples determined by DMA.

From the observed T_g_ values of samples 5 M, 11 M, 13 M, 14 M and 15 M, it is clear that the presence of crystallinity does not affect the resulting T_g_ values—see Figure 9. The differences that were found are within the measurement deviation. Although it is assumed that the temperature resistance also depends on the crystallinity of the sample, this was not confirmed for DGEBA/DGEBF mixtures containing semi-crystalline DGEBF [42]. T_g_ is one of the most correct indicators of cure of epoxy polymers and of their structural transformations [43].

### 4.8. Adhesion on Concrete

**Figure 10:**
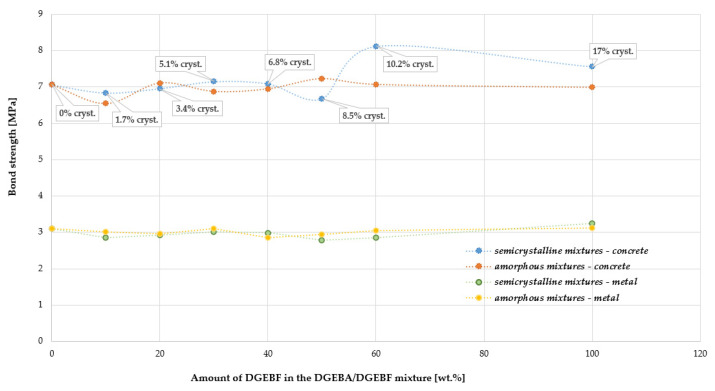
Results of the pull-off adhesion testing of the epoxy blends to concrete and metal. Dependence of stress at failure on crystallinity of DGEBF resin in different DGEBA/DGEBF resin mixtures.

From Figure 10 it can be seen that up to 10% crystallinity of the semi-crystalline mixtures, no significant differences in the adhesion of the epoxy samples to the concrete substrate can be observed. From 10% crystallinity onwards, a slight trend of increased adhesion can be found for the semi-crystalline mixtures, but in all cases, there was a failure in the concrete and so it is not possible to confirm this trend. However, it is evident that the adhesion of all epoxy mixtures tested to concrete was greater than 6.5 MPa. From Figure 10, no significant differences in the adhesion of the samples to the metal substrate can be observed. When testing the adhesion of the samples to metal, failure at the coating/metal interface was observed in all cases and the minimum adhesion was 2.8 MPa. Chain extension of liquid DGEBA epoxy with bisphenol A increased the bond strength of epoxy resin [43]. Prolongo et al. [44] found that the epoxy cured with aromatic amine presents a high adhesive strength and high T_g_.

### 4.9. Tensile Properties

**Figure 11:**
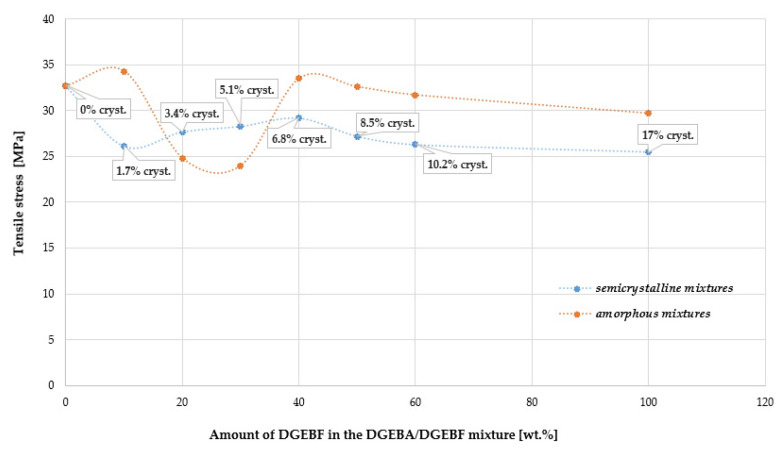
Comparison of the dependence of tensile stress at break σ_b_ on DGEBF content in amorphous and semi-crystalline DGEBA/DGEBF blends.

**Figure 12:**
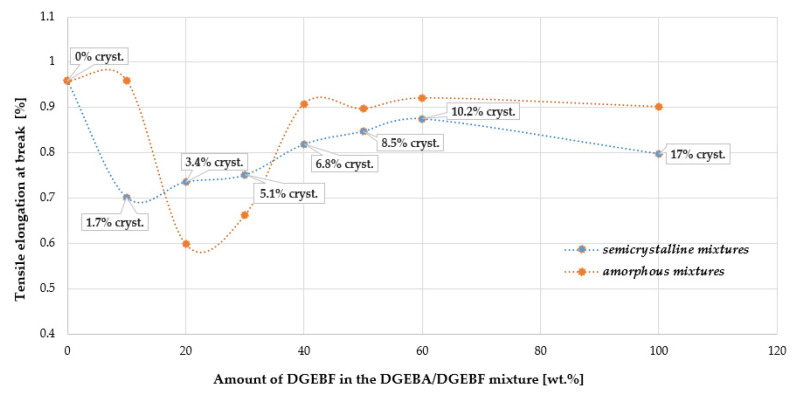
Comparison of the dependence of tensile strain at break σ_b_ on DGEBF content in amorphous and semi-crystalline DGEBA/DGEBF blends.

In Figure 11 and Figure 12, a decrease in tensile stress σ_b_ as well as a relative elongation ɛ_b_ (strain) can be observed at 20 to 30 wt.% DGEBF content in the amorphous DGEBA/DGEBF mixture. On the other hand, at DGEBF contents in the interval of 30 to 40 wt.%, the effect is reversed. The improvement in the relative elongation ɛ_b_ of the semi-crystalline polymer is evident at a DGEBF content of 20 to 60 wt.% in the crystallization phase “c” according to ISO 4897. Thus, at a certain crystallinity content in the blend, both mechanical parameters were improved. A slight strengthening effect of the crystalline phase at a crystallinity content of about 3 to 10% is evident in this case. Punchaipetch et al. [45] showed improvement in the tensile stress and strain at failure at low concentrations of diglycidyl ether of 4,4′-dihydroxybiphenol (DGE-DHBP) co-reacting in diglycidyl ether of bisphenol F (DGEBP-F). 

### 4.10. Chemical Resistance

**Figure 13:**
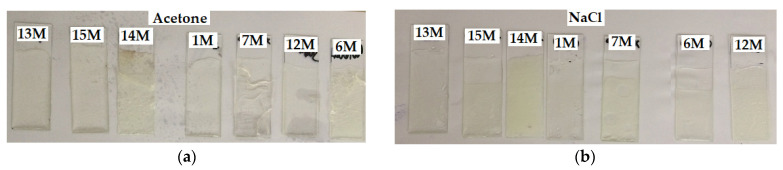

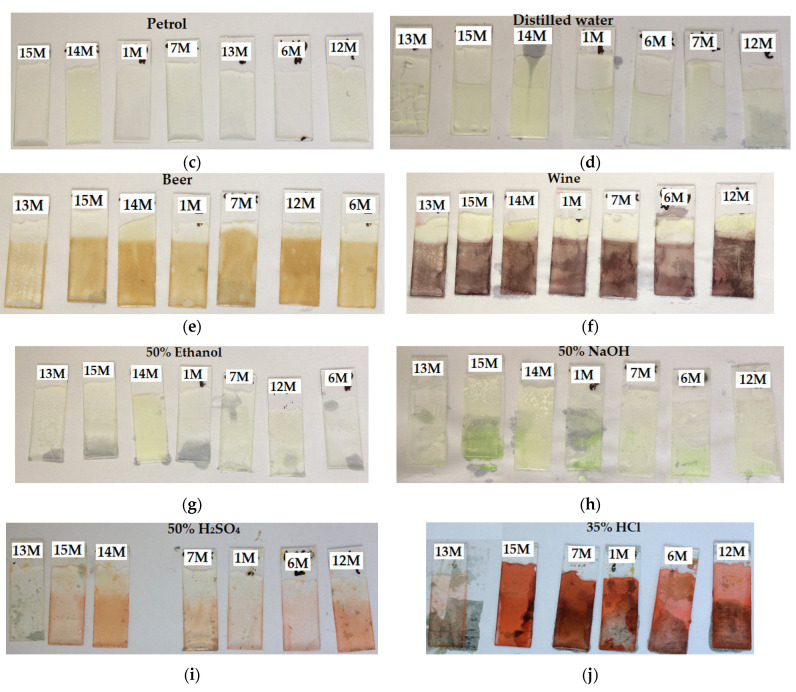
Samples after their exposure (immersion) to various chemically aggressive liquid environments: (**a**) acetone 1 day; (**b**) NaCl solution 1 day; (**c**) petrol 7 days; (**d**) distilled water 7 days; (**e**) beer 7 days; (**f**) wine 7 days; (**g**) 50% ethanol 7 days; (**h**) 50% NaOH 7 days; (**i**) 50% H_2_SO_4_ 7 days; (**j**) 35% HCl 7 days.

The evaluation of the chemical resistance in the form of weight changes in the samples is presented in Table 8 and the appearance of the samples after exposure to a chemically aggressive environment can be observed in Figure 13. The resistance to acetone was comparable for all tested samples—see Figure 13a. After 24 h, there were only very slight weight gains. The sample 14 M with the highest crystallinity showed the highest weight gain. After 7 days, all samples of epoxy systems immersed in acetone were already completely degraded.

The resistance to gasoline was very good in all samples. Weight gains were insignificant (completely zero for samples with a crystallinity up to 2%). The appearance of samples without crystallinity was constant throughout. Samples showing some degree of crystallinity gradually turned slightly yellow. 

Resistance to distilled water was worst for sample 14 M (highest crystallinity). Larger weight increments can be observed for samples with higher DGEBF content (amorphous and semi-crystalline) and for samples with higher crystallinity content. The appearance of the samples was already changed after 24 h of immersion. A dull map appeared on the surface of the samples, which slightly thickened during the rest of the test. The surface of sample 14 M (highest crystallinity) was significantly duller than the surface of the other samples after 4 weeks.

Resistance to beer and wine was comparable. Already after 24 h, the samples were discolored, at least for the samples with zero crystallinity. The intensity of discoloration increased steadily over time for all samples immersed in beer and wine. Samples immersed in beer first turned light yellow and then gradually orange. Samples immersed in red wine turned light pink after 24 h and gradually to deep red after 4 weeks. Samples immersed in beer and wine showed a dull surface after 24 h of immersion. Just as the coloration was strongest in the samples with the highest crystallinity, the weight increase was also highest in these samples.

Chemical resistance to 50% aqueous ethanol solution was found to be low for all samples and very low for samples with a crystallinity content ≤10% and for 15 M samples that contained only amorphous DGEBF. After 24 h of immersion, the samples began to yellow slightly. After 4 weeks of immersion, all samples were light yellow, and sample 14 M turned the most intense light yellow. The surface of the samples became darker.

In general, swelling, i.e., an increase in volume and weight of the sample, can be observed in samples containing solvents such as water, ethanol and acetone. With increasing crystallinity and increasing DGEBF content (amorphous or semi-crystalline), there was a decrease in the chemical resistance of the system. The good resistance to chemical attack is derived mainly from the aromatic nature of bisphenol A [46,47].

The resistance of the tested samples to saturated NaCl solution was very good. Again, a slight increase in weight can be observed after 4 weeks of immersion for samples with increasing crystallinity and increasing DGEBF content (amorphous or semi-crystalline). After 24 h of immersion, the samples started to turn slightly yellow (Figure 13b). After 4 weeks of immersion, all samples were pale yellow, and sample 14 M turned the most intense yellow. The surface of the samples became darker.

The chemical resistance of the samples to 50% aqueous NaOH solution was good. After 7 days, there was a slight weight loss. After 4 weeks, the weight loss was practically the same as after 7 days, but the samples had separated from the deposit. The coloration of the samples was slightly yellow with no major differences between samples—see Figure 13h. All samples except sample 13 M were matte.

Chemical resistance to acids was tested by immersion in 50% sulfuric acid and 35% hydrochloric acid. In both cases, there was a weight gain—see Table 9. In the case of hydrochloric acid weight gain occurred significantly, and in the case of sulfuric acid, only a slight weight gain occurred. For both acids, the trend observed for the previous chemicals was evident, namely that weight increases (and thus less chemical resistance) were observed for samples with higher crystallinity and higher DGEBF content (amorphous or crystalline) in the DGEBA/DGEBF mixture. The coloration of the samples for both acids was red. For hydrochloric acid, the coloration was more intense and the surface showed a coarser matte than for samples immersed in sulfuric acid. In the case of sulfuric acid, the 13 M sample was only slightly discolored. The surfaces of the samples exposed to both acids were matte.

## 5. Conclusions

In the research conducted, efforts were made to accelerate and retard crystallization more effectively, and the effect of crystallinity of bisphenol A- and bisphenol F-based epoxy resins on the resulting parameters of hydrophobic coating systems based on these resins was investigated. These materials are used already during construction as preventive protection, but also during reconstruction and repair as behavioral and hydrophobizing materials. They can fulfill not only a protective function, but also an aesthetic and technical function, for example in industrial flooring. The influence of the filler surface structure on the crystallization rate has been demonstrated. The fillers and thixotropic additives used at lower dosages with particle sizes up to 63 μm accelerated the crystallization process to the extent that the samples reached crystallization stage “b” within 28 days of the start of the tests. The crystallization process of the samples containing auto-glass was significantly slower—a smoother particle surface structure and on the fracture surfaces. As a result of this fact, the efficiency of glass particles as crystallization nuclei is significantly lower. The dependence of the crystallization process, or nucleation rate, on the specific surface area was also confirmed. From the results, it is clear that both homogeneous and heterogeneous inoculations are more effective methods of accelerating the crystallization process than the addition of nucleation additives. Homogeneous inoculation of the “microseeds” type applied to the DGEBF resin appears to be the fastest way to accelerate the crystallization process.

The presence of crystallinity was found to increase the hardness and brittleness of the resulting polymer. However, an exception was found. The results obtained provide valuable information on the effect of crystallinity at around 5% (up to 10% for some types of stress) on the mechanical parameters. A system with a crystallinity of about 5% contains mainly small crystals. A system containing small crystals in a low concentration does not follow the behavior of composite materials that occurs at crystallinities of about 10% and higher. In such a system, the mechanical behavior of the interphase between the amorphous and the crystalline phase is applied, which occupies a significantly larger volume of the matrix than the crystalline phase. This intermediate phase is formed by the so-called crystalline phase. The interfacial phase is formed by “polymer tie molecules” which are partially incorporated into the crystal but the bulk of which is amorphous. However, the interphase behaves neither as an amorphous phase nor as a crystalline phase. A characteristic of this interphase is a greater tendency to deform than that of the amorphous and crystalline phases. For this reason, at a crystallinity of about 5%, the systems studied show a fluctuation in the determined mechanical parameters.

The research carried out in the field of crystallization of epoxy coating systems brings with it a better orientation in the problem of crystallization of coating and hydrophobic systems based on epoxy resins and an improvement in the state of existing knowledge.

## Figures and Tables

**Table 1:** Properties of the unmodified epoxy resins based on bisphenol A (DGEBA) and bisphenol F (DGEBF) [27,28].

Parameter	DGEBA	DGEBF
Epoxide equivalent weight (EEW) (g/eq)	182–192	167–174
Viscosity (25 °C) (mPa·s)	11,000–14,000	3400–4200
Density (25 °C) (g/cm^3^)	1.16	1.19
Hydrolysable chloride (ppm)	<500	<1000

**Table 2:** Properties of the fillers.

Type of Filler	Producer	Max. Particle Size (μm)	Particle Content > 63 μm (%)	Specific Gravity (kg/L)
Baryte	Koltex Color	63	–	4.5
Waste windshield glass	–	63	–	2.5
Silica flour Dorsilit 1600	Dorfner	160	36	2.6
Silica flour Dorsilit 12100	Dorfner	63	–	2.6

**Table 3:** Properties of the reactive and non-reactive diluents.

Type of Solvent	Solvent	Viscosity at +25 °C (mPa·s)	Boiling /Flash Point (°C)	Density at +25 °C (g/cm^3^)
Reactive	Epilox P 13–18	4–12	>200/142	0.89
Reactive	Araldite DY-E	5–10	>200/142	0.89
Non-reactive	Benzyl alcohol	6.6	205/101	1.045
Non-reactive	Methylisobutylketon	0.6	117/16	0.802

**Table 4:** Sample formulations for the determination of tendency to crystallize according to ISO 4895 standard.

Sample	Component	Amount (g)	Nucleating Agent	Amount (g)
1 N	DGEBA	20	CaCO_3_	20
			Ethanol	2
2 N	DGEBF	20	CaCO_3_	20
			Ethanol	2
3 N	DGEBA	14	CaCO_3_	20
	Epilox P 13–18	6	Ethanol	2
4 N	DGEBA	14	CaCO_3_	20
	Araldite DY-E	6	Ethanol	2
5 N	DGEBA	14	CaCO_3_	20
	benzyl alcohol	6	Ethanol	2
6 N	DGEBA	14	CaCO_3_	20
	methylisobutylketon	6	Ethanol	2
7 N	DGEBA	19	CaCO_3_	20
	PS 200 m^2^/g	1	Ethanol	2
8 N	DGEBA	19	CaCO_3_	20
	PS 380 m^2^/g	1	Ethanol	2
9 N	DGEBA	10	CaCO_3_	20
	windshield glass	10	Ethanol	2
10 N	DGEBA	15	CaCO_3_	20
	baryte	5	Ethanol	2
11 N	DGEBA	10	CaCO_3_	20
	silica flour 1600	10	Ethanol	2
12 N	DGEBA	15	CaCO_3_	20
	silica flour 12,100	5	Ethanol	2

**Table 5:** Formulations for the determination of the mechanical parameters, chemical resistance and glass transition temperature of chosen epoxy systems with a certain degree of crystallinity.

Sample	Components	Amount (g)
1 M	DGEBA amorphous	180
	DGEBF amorphous	20
	Isophoronediamine-based hardener	102
2 M	DGEBA amorphous	160
	DGEBF amorphous	40
	Isophoronediamine-based hardener	103
3 M	DGEBA amorphous	140
	DGEBF amorphous	60
	Isophoronediamine-based hardener	103
4 M	DGEBA amorphous	120
	DGEBF amorphous	80
	Isophoronediamine-based hardener	104
5 M	DGEBA amorphous	100
	DGEBF amorphous	100
	Isophoronediamine-based hardener	105
6 M	DGEBA amorphous	80
	DGEBF amorphous	120
	Isophoronediamine-based hardener	106
7 M	DGEBA amorphous	180
	DGEBF in the crystallization phase “c”	20
	Isophoronediamine-based hardener	102
8 M	DGEBA amorphous	160
	DGEBF in the crystallization phase “c”	40
	Isophoronediamine-based hardener	103
9 M	DGEBA amorphous	140
	DGEBF in the crystallization phase “c”	60
	Isophoronediamine-based hardener	103
10 M	DGEBA amorphous	120
	DGEBF in the crystallization phase “c”	80
	Isophoronediamine-based hardener	104
11 M	DGEBA amorphous	100
	DGEBF in the crystallization phase “c”	100
	Isophoronediamine-based hardener	105
12 M	DGEBA amorphous	80
	DGEBF in the crystallization phase “c”	120
	Isophoronediamine-based hardener	106
13 M	DGEBA amorphous	200
	Isophoronediamine-based hardener	101
14 M	DGEBF in the crystallization phase “c”	200
	Isophoronediamine-based hardener	109
15 M	DGEBF amorphous	200
	Isophoronediamine-based hardener	109

**Table 6:** Chemical aggressive media used for the chemical resistance test of the epoxy blends.

Type of Chemical	Concentration (%)
Acetone	100
Technical petrol	100
Distilled water	100
Beer	100
Wine	100
Aqueous NaCl solution (saturated)	27
Aqueous NaOH solution	50
Aqueous HCl solution	35
Aqueous H_2_SO_4_ solution	50
Aqueous solution of ethanol	50

**Table 7:** Results of the analysis of the tendency to crystallization—28 days 2 times per day.

Sample	1/M ^1^–4/A ^2^	5/M–13/A	14/M–19/A	20/M–A	21/A–25/A	26/M–28/M	28/A
1 N	a	a	b	b	b	b	b
2 N	a	b	b	c	c	c	c
3 N	a	a	a	a	a	a	a
4 N	a	a	a	a	a	a	a
5 N	a	a	a	a	a	a	a
6 N	a	a	a	a	a	a	a
7 N	a	a	a	a	a	b	b
8 N	a	a	a	a	b	b	b
9 N	a	a	a	a	a	a	b
10 N	a	a	a	a	a	a	b
11 N	a	a	a	a	a	a	a
12 N	a	a	a	a	a	a	b

^1^ M = 7 a.m., ^2^ A = 5 p.m.

**Table 8:** Crystallinity of resins for the sample preparation.

The Crystalline Phase of the Sample	Specific Gravity (kg/L)	Calculated Crystallinity (%)
DGEBF amorphous	1.1952	–
DGEBF in the crystallization phase “c”	1.1960	17
DGEBF maximally spontaneously crystallized	1.1999	–

**Table 9:** Results of the chemical resistance test—change in sample weight in (g).

Sample/ Crystallinity	Time of Exposure (Days)	Acetone	Petrol	Distilled Water	Beer	Wine	NaCl Solution	50% NaOH	35% HCl	50% H_2_SO_4_	50% Ethanol
1 M/0	1	+0.03	+0.00	+0.00	+0.00	+0.00	+0.00	−0.00	+0.01	+0.00	+0.00
	7	N/A *	+0.00	+0.05	+0.01	+0.02	+0.00	−0.02	+0.06	+0.02	+1.25
	28	N/A	+0.01	+0.95	+0.21	+0.24	+0.01	−0.03	+0.68	+0.21	+5.01
6 M/0	1	+0.05	+0.00	+0.00	+0.00	+0.00	+0.00	−0.00	+0.02	+0.00	+0.00
	7	N/A	+0.00	+0.13	+0.05	+0.09	+0.00	−0.03	+0.08	+0.07	+2.02
	28	N/A	+0.03	+1.07	+0.32	+0.36	+0.03	−0.04	+0.71	+0.28	+6.23
7 M/1.7	1	+0.06	+0.00	+0.00	+0.00	+0.00	+0.00	−0.00	+0.02	+0.00	+0.00
	7	N/A	+0.00	+0.07	+0.02	+0.02	+0.00	−0.03	+0.08	+0.04	+1.33
	28	N/A	+0.02	+0.99	+0.19	+0.20	+0.01	−0.03	+0.70	+0.23	+5.21
12 M/10.2	1	+0.06	+0.00	+0.00	+0.00	+0.00	+0.00	−0.00	+0.02	+0.00	+0.00
	7	N/A	+0.00	+0.21	+0.07	+0.11	+0.00	−0.04	+0.11	+0.10	+3.84
	28	N/A	+0.03	+1.34	+0.45	+0.50	+0.04	−0.05	+0.76	+0.31	+8.97
13 M/0	1	+0.00	+0.00	+0.00	+0.00	+0.00	+0.00	−0.00	+0.02	+0.00	+0.00
	7	N/A	+0.00	+0.04	+0.02	+0.02	+0.00	−0.01	+0.07	+0.02	+1.18
	28	N/A	+0.00	+0.84	+0.05	+0.07	+0.00	−0.02	+0.67	+0.19	+4.96
14 M/17	1	+0.11	+0.00	+0.00	+0.00	+0.00	+0.00	−0.00	+0.04	+0.00	+0.00
	7	N/A	+0.00	+0.53	+0.32	+0.37	+0.00	−0.06	+0.11	+0.14	+5.67
	28	N/A	+0.02	+2.41	+0.98	+1.03	+0.03	−0.07	+0.78	+0.43	+11.83
15 M/0	1	+0.06	+0.00	+0.00	+0.00	+0.00	+0.00	−0.00	+0.02	+0.00	+0.00
	7	N/A	+0.00	+0.23	+0.12	+0.09	+0.00	−0.03	+0.07	+0.09	+2.46
	28	N/A	+0.00	+1.31	+0.45	+0.39	+0.00	−0.04	+0.68	+0.32	+7.78

* Not applicable—samples were damaged after exposure to acetone.

## Data Availability

Not applicable.
